# The Reform of Medical Education in France

**Published:** 1911-01-28

**Authors:** 


### The Reform of Medical Education in France.

The present situation in France, so far as the medical schools are concerned, is worth earnest study by medical educationists in this country. Elsewhere in this issue we give an abstract of Professor Leredde's able exposition of the case against excessive centralisation, and it is perhaps well to remind our readers that the arguments against the existing French system do not apply to our schools beyond a certain point. In the matter of preliminary scientific education the French medical student is in a far better position than his English colleague. For the former, centralisation means the best teaching by the best masters—teachers, be it understood, who are not merely great scientists but great masters as well, and who have the gift of attracting disciples. Our system, on the other hand, means the distribution of various teaching units, of varying degrees of value, at scattered centres, where their activities are dissipated upon small classes in a haphazard fashion. The fact that, as someone has recently declared, we have so few scientific medical men or medical scientists is entirely due to the absurd fashion in which the medical schools of London persist in attempting the impossible task of establishing microscopic universities in various parts of the Metropolis.

But it is when excessive centralisation touches the actual subjects of the medical curriculum that it becomes indefensible. In France, and to some extent in Italy and Austria, it has done so. In Germany, on the other hand, the peculiarly effective provisions which have been made at all the large teaching centres have tended to diminish the bad effects of such centralisation to a large degree. With us there is no centralisation, and our practical medical education is therefore in many ways as excellent as it can be made. Where it fails, it fails through causes which are extraneous to the system —the want of centralisation in the preliminary studies, the unfortunate conditions at some medical schools and hospitals, and so on. Perhaps it is unlikely that our system has benefited medicine to the same extent as the German and French systems have. We have produced no "schools" in the best sense of the term, and though individuals have gained a reputation as teachers with us, we have no Virchow or Bergmann, no Charcot or Charpentier or Pirogoff—men, that is, who have influenced the whole trend of medical teaching in their respective countries. Even in a microcosm like London there is already a blurring of individuality; to average medical students who have not had the privilege of walking the wards of University, Gowers (to take only one instance at haphazard) is a name merely, the writer of a text-book which it is unnecessary to read up for the Final. In Berlin, on the contrary, every medical student knows Krause personally, and has the chance of knowing him intimately—and that notwithstanding the fact that there are more medical students there than in London. Similarly, in Paris every student knows Dieulafoy, Landouzy, Delepine—every one, in fact, of the phalanx of professors attached to the University, no matter whether they are active at hospitals which lie miles apart from each other. On the other hand, the medical student in this country has larger scope for initiative and originality. He has greater difficulties to contend with in practical work, greater responsibilities are thrust upon him even before he is qualified, and the results show that he is on the whole able to justify the trust that our system puts in his determination and capacity. We are still struggling to find the ideal in medical education. During the next ten years the curriculum will doubtless be chopped and changed, just as it has been altered and amended during the last decade. In view of this possibility, it is worth while to pay some attention to the developments in other countries and to test the value of our system by comparison with methods that prevail elsewhere.

